# Resolution of Postsurgical Diplopia, Paresthesia, and Weakness Following Inpatient Massage Therapy: A Case Report

**DOI:** 10.1177/2164956119852396

**Published:** 2019-05-16

**Authors:** Jennifer Hauschulz, Stephanie Clark, Brent Bauer, Tony Chon

**Affiliations:** 1 Integrative Medicine and Health, Mayo Clinic, Rochester, Minnesota

**Keywords:** massage therapy, integrative medicine, pain

## Abstract

Massage therapy is utilized in hospitals for patients experiencing pain, anxiety, sleeplessness, among other symptoms. Postsurgical pain is one of the most common reasons massage therapy is ordered. We present a case of a 45-year-old woman who underwent ventral hernia repair. Postoperative day 2, she began to experience multiple symptoms, including double-vision, left-sided facial numbness, tremors, pain, and weakness in her left fingers, arm, and leg. A static magnetic resonance image of the brain and cervical spine were obtained, which revealed disc protrusions at C3/C4, C5/C6, C6/C7, and mild deformity of the spinal cord. The patient’s pain was difficult to control and she was unable to be weaned from intravenous pain medication. Massage therapy was ordered on postoperative days 6 and 7. During both massage sessions, as upper neck muscle tension was reduced, the therapist and patient observed several audible “pops” of the cervical spine with immediate relief of symptoms. In this particular case, massage therapy, though requested to address pain, had a secondary benefit in relieving her diplopia, left-sided facial numbness, tremors, and weakness. Although the mechanism of action is not clear, this case highlights the significant secondary beneficial effects that often occur with massage therapy.

## Introduction

Patients undergoing surgery are often faced with a number of challenges in the postoperative setting, including pain, nausea, and anxiety.^1,2^ Pain is a particularly common occurrence in the postoperative treatment^1–3^ and is one of the most common reasons massage therapy is requested in the postoperative setting. In addition to pain, surgical patients can experience a number of secondary symptoms, often times caused or contributed to by prolonged positioning during surgery. Previous studies^4,5^ have identified uncommon, but challenging symptoms, such as diplopia, vertigo, weakness, and parasthesias^4,5^ that may occur post procedure due to positioning of the cervical spine.^4^ Secondary symptoms can be a manifestation of anxiety;^6^ regardless of cause, they can be difficult to treat. In the present case, we suggest that massage therapy, using Swedish massage, while primarily directed at treating pain, may have provided secondary, but significant improvement of diplopia, paresthesia, pain, and weakness.

## Presenting Concerns

The patient was a 45-year-old woman admitted to the hospital for an elective, ventral hernia repair in 2018. The operative course was described as uncomplicated. Following surgery, the patient was admitted to Trauma-Critical Care-General Surgery service for continued postoperative care. Unfortunately, by postoperative day 2, her stay was complicated by the development of intractable pain in left forearm and third, fourth, fifth fingers, diplopia, and vertigo, paresthesia involving her face and left upper extremity, and weakness in her left leg. Brain and cervical spine magnetic resonance imaging (MRIs) were obtained with and without contrast to further evaluate her symptoms. The findings noted mild straightening and loss of cervical lordosis and mild multilevel disc desiccation. It was also noted mild deformity of the cord by disc protrusion at C6/C7, left of the midline and mild to moderate compromise of the exiting nerve roots on the right side of C5/C6 and C3/C4. Both Neurology and Ophthalmology services were consulted. The neurology evaluation did not demonstrate any objective findings to explain her symptoms. The ophthalmology evaluation took place after massage therapy, which confirmed her diplopia had resolved.

## Clinical Findings

On postoperative day 6, massage therapy was ordered to help mitigate the patient’s pain. When seen by the massage therapist, the patient had stable vital signs and appeared alert; however, she was wearing an eye patch, which according to the Eye Institute^7^ covering one eye can tend to help with diplopia. The patient reported pain 8/10 and had been receiving multiple forms of pain control including, fentanyl, acetaminophen, gabapentin, baclofen, diazepam, hydromorphone, and bupivacaine thoracic epidural, without significant benefit. The patient was still experiencing intermittent diplopia, left-sided paresthesia and weakness, and ringing and “pressure” in both ears.

## Therapeutic Focus and Assessment

### First Massage Therapy Session

On the sixth postoperative day, at 14:00, an inpatient massage therapist arrived and assessed the patient. The patient reported pain in the left side of her neck, occiput, and shoulder, with tingling and “jumping” of left shoulder, numbness in face, hands, and left leg, ear pressure, and diplopia.

Patient received a 25-minute massage, while seated in her hospital chair. Swedish massage techniques, effleurage, petrissage, and friction, were provided with focus to the left upper, middle back, neck, occiput, and shoulder. Upon palpation, moderate tension was noted on the left suboccipitals, cervical and thoracic paraspinal, levator, scalene, and upper, middle, and lower trapezius. Throughout the massage, there were several audible “pops” attributed to adjustments of the cervical spine. The patient’s head would abruptly shift as the spine adjusted. At this time, the therapist would stop the massage and have the patient rotate her neck and report how she was feeling. Following these “pops,” the patient immediately reported relief of ear and eye pressure and blurriness and reduced facial and arm paresthesia. Post massage therapy, the patient noted that she was not experiencing headaches, diplopia, and left-sided numbness. At 16:30, she was evaluated by a physician who documented the patient reported her symptoms had improved; and her only complaint was right-sided neck tightness.

### Second Massage Therapy Session

On the seventh postoperative day, at 11:00, the same massage therapist returned. The patient requested massage to her right neck and shoulder, to address muscle tension. Upon palpation, moderate tension was noted of the right levator, scalene, and bilateral cervical and thoracic paraspinal. Swedish massage techniques provided immediate tension release, particularly of the suboccipitals, scalene, levator, thoracic, and cervical paraspinal and trapezius. Throughout the massage, the patient reported a “popping” sound in her right ear and of relief of pain.

## Follow-up and Outcomes

**Figure 1.**
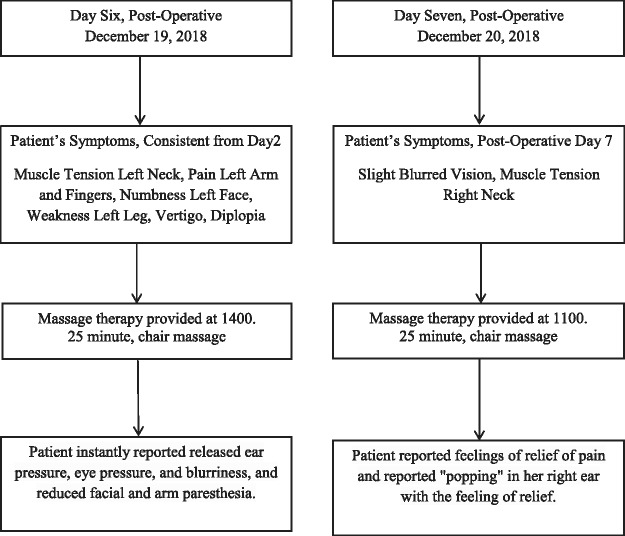
Postoperative timeline of massage therapy sessions.

The patient received 2 massage therapy sessions, see Figure 1. Following the first treatment, the patient reported complete alleviation of her diplopia and a marked reduction in pain on the left neck pain, occiput, and shoulder. She also reported an immediate reduction of facial and arm numbness and reduced ear and eye pressure. However, the patient still was experiencing right-sided muscle tightness and slight blurred vision. The following day, during the second session, the patient reported relaxed muscles and relief from the blurred vision.

Following massage therapy, the patient’s narcotic requirements were reduced; she was successfully weaned off the fentanyl patient‐controlled analgesia and discontinued the diazepam. She was discharged on postoperative day 8. She had no further recurrence of her diplopia, paresthesia, and weakness, and this was noted to still be true at her outpatient follow-up 1 week later and during an additional follow-up on January 3, 2019. She was readmitted to the hospital approximately 3 weeks later for postoperative wound infection and requested massage therapy. She reported to the therapist that her previous symptoms had not returned.

## Discussion

The present case describes the clinical course of a patient who underwent elective, surgical repair of ventral hernia, and then developed several postoperative complications, including diplopia, paresthesia, pain, and weakness. MRI findings demonstrated mild findings of disc protrusion and mild, contralateral compromise of C3/C4 and C5/C6. However, observation of mild straightening, loss of cervical lordosis, and mild multilevel disc desiccation was present. Further assessment with Neurology resulted in no objective findings to explain her symptoms. As noted, published reports have highlighted the effects of dynamic, direct mechanical compression of the upper cervical spinal cord, causing symptoms of brainstem compromise in the absence of significant radiographic evidence of osseous instability. In addition, previous reports indicate that dynamic cervical MRI can show a number of findings in comparison to static cervical MRI.^8^ It is unclear whether a dynamic cervical MRI imaging may have noted the dynamic changes related to spinal misalignment and contributions of osseous or muscular structures.

Although massage therapy was ordered to help mitigate pain, on postoperative days 6 and 7, the techniques provided on her neck and shoulders resulted in acute and significant improvement of her symptoms, with no recurrence. Patient was subsequently discharged from the hospital on the following day. Limitations of this report include the fact that massage therapy was not ordered for the patient until days 6 and 7 of the patient’s stay. Another limitation is the Ophthalmology examination took place after massage when the diplopia was already resolved so we were unable to use their expertise in this case. Future studies should include a massage therapy consult with the patient earlier on during hospital stay and radiology involvement pre and post massage therapy including dynamic cervical MRI imaging.

In a review of the literature, there was a case study examining 4 different cases of massage therapy and its effects on osteoarthritis, scoliosis, spinal stenosis, and degenerative disc disease.^9^ While successful at improving pain, this was noted more in the lower back and not in the cervical vertebrae, as seen in this case study. In addition, this case is unique in the fact that a previous study on the cervical vertebrae^10^ only focused on range of motion and coupled it with chiropractic work. Although the etiology of the diplopia, paresthesia, and weakness were unclear from Neurology’s point of view, the symptoms may have been connected to the cervical vertebra being out of alignment leading to symptoms as described with subsequent resolution following massage therapy dedicated to the neck and shoulders. This case report suggests that further investigation is needed when other patients present with similar scenarios, consider dynamic brainstem compression as an etiology of symptoms from spinal misalignment, consider modification of imaging techniques when dynamic changes may be suspected with no objective findings, and continue to investigate the benefits of nonpharmacologic therapy for pain management and patient care.

## Patient Perspective

Jennifer was able to relieve my numbness in my face and shooting pain and numbness in my arm and fingers. She was able to relieve my blurry and double vision. This was all after my first session. My second session I had the pressure from behind both eyes go away and dizziness go away. After the second session, the ringing in my ears went away and was able to watch TV and walk without any issues. Thanks to the massage my dizziness and nausea went away and today is day 3 after starting massage and no return. Thank you very much for your service and quick response and helping me with my recovery.

## References

[1] Cutshall SM Wentworth LJ Engen D Sundt TM Kelly RF Bauer BA. Effect of massage therapy on pain, anxiety, and tension in cardiac surgical patients: a pilot study. Complement Ther Clin Pract. 2016; 16(2):92–95. 10.1016/j.ctcp.2009.10.006 20347840

[2] Dreyer NE Cutshall SM Huebner M et al Effect of massage therapy on pain, anxiety, relaxation, and tension after colorectal surgery: a randomized study. Complement Ther Clin Pract. 2015; 21(3):154–159. 26256133 10.1016/j.ctcp.2015.06.004

[3] Vergo MT Pinkson BM Broglio K Li Z Tosteson TD. Immediate symptom relief after a first session of massage therapy or Reiki in hospitalized patients: a 5-rear clinical experience from a rural academic medical center. J Altern Complement Med. 2018; 24(8):801–808. 29620922 10.1089/acm.2017.0409 PMC6422004

[4] Kamel I Barnette R. Positioning patients for spine surgery: avoiding uncommon position-related complications. World J Orthop. 2014; 5(4):425–443. 25232519 10.5312/wjo.v5.i4.425 PMC4133449

[5] Rosenberg WS Salame KS Shumrick KV Tew JM Jr. Compression of the upper cervical spinal cord causing symptoms of brainstem compromise. A case report. Spine. 1998; 23(13):1497–1500. 9670404 10.1097/00007632-199807010-00013

[6] Caykoylu A Ekinci O Albayrak Y Kuloglu M Deniz O. Arnold–Chiari I malformation association with generalized anxiety disorder: a case report. Prog Neuropsychopharmacol Biol Psychiatry. 2008; 32(6):1613–1614. 18579276 10.1016/j.pnpbp.2008.05.018

[7] Eye Institute. *Double Vision* https://www.eyeinstitute.co.nz/About-eyes/A-to-Z-of-eyes/Symptoms/Double-Vision. Published 2019. Accessed April 4, 2019.

[8] Nigro L Donnarumma P Tarantino R Rullo M Santoro A Defini R. Static dynamic cervical MRI: two useful exams in cervical myelopathy. J Spin Surg. 2017; 3(2):212–216. 10.21037/jss.2017.06.01 PMC5506301 28744502

[9] Allen L. Case study: the use of massage therapy to relieve chronic low-back pain. Int J Therap Massage Bodywork. 2016; 9(3):27–30. 27648110 10.3822/ijtmb.v9i3.267 PMC5017818

[10] Avery RM. Massage therapy for cervical degenerative disc disease: alleviating a pain in the neck? Int J Ther Massage Bodywork. 2012; 5(3):41–46. 23087777 10.3822/ijtmb.v5i3.146 PMC3457722

